# Bath and Her Thermal Waters

**Published:** 1889-12

**Authors:** 


*Bath and her Thermal Waters.* By SAMUEL CRADDOCK, M.R.C.S. *Bath Chronicle* Office. 1889.

The literature of English spas has hitherto been meagre in the extreme; but Bath has recently done its best to make up for the deficiency. Book after book has emanated from that city, each in its turn giving an account of the history of the city and its baths, its climate, and the advantages to the invalid from a residence within it. This, the latest of the series, differs from its predecessors, inasmuch as it endeavours to sift what the author believes to be the chaff from the wheat, "its object being to endeavour to define, categorically, the real therapeutic value of the Waters." This endeavour to "place medical treatment by the Bath Waters on a rational and incontrovertible basis" concludes with the following deductions:

1. That the diseases in which the Bath Waters are of advantage are exceedingly limited in number.

2. The value of these waters is much increased when used in conjunction with appropriate medicines.

3. Gout, in all its phases, is the one disease in which this action may be considered specific, and by periodical treatment effete materials in the system that tend to cause organic changes in the heart, kidneys, and even the brain are combated; whilst the intervals of attack are not only widened, but the tenure of life is proportionately prolonged.

4. In chronic muscular rheumatism the excellent bathing arrangements are of much value.

5. In rheumatoid arthritis great care should be taken in the selection of cases; whilst those of a decided neurotic origin should be positively excluded.

6. Cases of lead-palsy may also use the waters to advantage.

The author's experiments in the elimination of uric acid are of much interest: he shows that drinking the waters and immersion in the waters increases the excretion of uric acid, whilst the urea is proportionately diminished.

